# Inner Retinal Layer and Outer Retinal Layer Findings after Macular Hole Surgery Assessed by means of Optical Coherence Tomography

**DOI:** 10.1155/2019/3821479

**Published:** 2019-04-01

**Authors:** Maria Vittoria Cicinelli, Alessandro Marchese, Francesco Bandello, Michele Coppola

**Affiliations:** ^1^Department of Ophthalmology, Università Vita-Salute, IRCCS Ospedale San Raffaele, Milan, Italy; ^2^Ophthalmology Unit, Azienda Ospedaliera di Monza, Monza, Italy

## Abstract

**Aim:**

To summarize the spectrum of optical coherence tomography (OCT) and OCT angiography (OCTA) features after full-thickness macular hole (MH) repair surgery.

**Methods:**

A PubMed engine search was carried out using the terms “Macular Hole,” “Optical Coherence Tomography,” and “Optical Coherence Tomography Angiography.” All reports published in English up to October 2018, irrespective of their publication status, were included. Tomographic signs analyzed were divided according to the involved portion of the retina in “inner retinal layers” and “external retinal layers.” Despite predominantly involving the inner retinal layers, cystoid macular edema (CME) has been treated as a separate entity. Finally, report on vessel density (VD) changes and the foveal avascular zone (FAZ) area modifications have been included.

**Results:**

Different clinical findings can be observed on OCT of patients who underwent MH repair surgery. There is general consent that retinal thinning involving primarily the retinal nerve fiber layer and the ganglion cell layer takes place after surgery. In the postoperative period, the outermost retinal layers get progressively restored. Persistent defects in the ellipsoid zone or in the external limiting membrane correlate with worse postoperative visual outcome. OCTA has globally demonstrated that eyes after MH closure show a reduction in macular and paramacular VD and smaller FAZ areas, compared with control or fellow eyes.

**Conclusion:**

Clinicians should be aware of the most common tomographic findings to properly manage each condition. In addition, significant advantages for the postoperative application of OCT and OCTA include noninvasiveness, rapid and simple execution, repeatability, and precise measurements.

## 1. Introduction

Vitreoretinal surgery has been an object of great innovation during the last years. New intraoperative instrumentation, such as vitrectomy probes [1, 2], illumination techniques [3], and wide-angle viewing systems, has increased the safety, the effectiveness, and the repeatability of the surgical maneuvers. On the other hand, the usage of noninvasive imaging techniques, such as optical coherence tomography (OCT), has enhanced the detection of many subtle vitreal, retinal, and choroidal changes, which are difficult or impossible to be visualized by indirect ophthalmoscopy [4].

The application of the OCT and its newest developments, such as enhanced-depth imaging (EDI) [5] or swept-source OCT [6], has widened the spectrum of vitreoretinal conditions, leading to the introduction of new clinical entities and the better understanding of the traditional ones, including macular hole (MH).

The preoperative assessment of MH by means of OCT is fundamental for the evaluation of several important features that have been recognized to contribute to the anatomical and functional outcome after surgical repair. The noninvasive morphological investigation of these lesions has allowed for the fundamental distinction between full-thickness macular hole (FTMH) [7], characterized by an interruption in the neuroretina involving all the sensory layers, and lamellar MH (LMH) [8]. LMH, in turn, can be divided into degenerative and tractional, on the bases of specific morphologic features. The former is characterized by the presence of a foveal bump, lamellar hole-associated proliferation, a disrupted ellipsoid zone in the large majority of the cases, and a round-edged intraretinal cavitation involving outer retinal layers. The latter is almost invariable associated with the presence of tractional epiretinal membrane, an intact ellipsoid layer, and a sharp-edged schisis between the outer plexiform and the outer nuclear layer [9]. According to some authors, tractional LMH should be considered as being part of the pseudohole category with stretched foveal edges [10].

In the recent years, a new OCT-based classification of MH has been published by the International Vitreomacular Traction Study (IVTS) Group (Table 1) [11]. This classification accounts for the size, the cause, and the vitreous state at the macular region, known as vitreomacular interface (VMI), and its interaction with the neuroretinal layers and is one of the leading criteria driving the therapeutic approach to MHs.

Thanks to the recently published pieces of evidence, several OCT features have been identified as important prognostic parameters to be considered in the surgical planning, including the presence of an epiretinal membrane (ERM) or a lamellar hole-associated epiretinal proliferation (LHEP) [12, 13] and the state of the internal or external retinal layers [14].

Besides preoperative OCT studies, the intraoperative and postoperative monitoring of the surgical outcomes has been giving unattended insights on the response of the retinal tissue to the closing procedures [15, 16]. In fact, biomicroscopy is seldom able to evaluate the extent of retinal morphologic changes that take place after the surgery and is not able to establish any correlation between the anatomical and functional findings. On the other hand, visual symptoms are too dependent on patients' subjective perception and their psychophysical state to become a reliable parameter for the surgical outcome.

Conversely, postoperative OCT has demonstrated undeniable ability to capture microscopic anatomical details. In fact, especially in cases in which vitreal substitutes, like silicone oil or expansible gas, impair the view of the posterior pole, noninvasive imaging monitoring of the macula is a precious instrument for patients' evaluation and follow-up. Qualitative assessment of the OCT includes the analysis of the different retinal structures, the remnants of the ERM and the internal limiting membrane (ILM), and the release of any point of vitreomacular attachment (VMA) or vitreomacular traction (VMT). In parallel, quantitative evaluation relies on standard objective parameters offered by the modern OCT software, including the central macular thickness (CMT), retinal nerve fiber layer (RNFL), ganglion cell layer (GCL), and inner plexiform layer (IPL) thickness. Finally, the introduction of OCT angiography (OCTA) [17], a relatively new, dyeless, depth-resolved technique that allows the visualization of retinal microvasculature by detecting intravascular blood flow, has been used to noninvasively investigate retinal capillary networks and foveal avascular zone (FAZ) changes in patients who underwent macular surgery.

The aim of this review is to summarize the spectrum of OCT features after MH repair surgery, focusing on the prognostic signs a retinal surgeon should be aware of in order to predict the final visual outcome.

## 2. Methods

A PubMed engine search was carried out using the terms “Macular Hole,” “Lamellar Macular Hole,” “Optical Coherence Tomography,” and “Optical Coherence Tomography Angiography.” All reports published in English up to October 2018, irrespective of their publication status, were reviewed. From the results, articles were selected based on the degree of relevance as determined by the authors: original studies were preferred; case reports or case series were included only if deemed of particular interest. The majority of the articles taken into consideration were referred to FTMH. However, due to paucity of articles published so far about OCTA in MHs, data from LMH were also included in the review.

**Figure 1:**
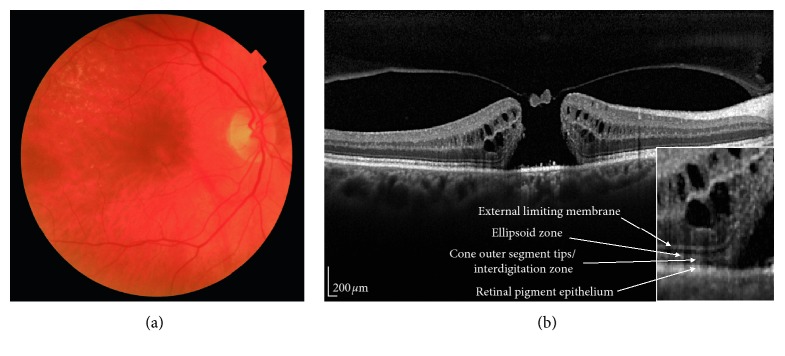
A spectral domain optical coherence tomography (SD-OCT) scan passing through the fovea in a patient with a large macular hole. (a) Color fundus of the patient; (b) SD-OCT scan showing a small operculum on the roof of the macular hole. Bottom right: magnification of the scan showing a detail of the four external retinal layers.

Tomographic signs analyzed in this review were divided according to the involved portion of the retina; “inner retinal layers” included all the structures between the ILM and the external limiting membrane (ELM) (RNFL, GCL, and IPL). On the contrary, “external retinal layers” included the structures comprised between the ELM and Bruch's membrane, namely, the four hyper-reflective outer lines on SD-OCT: the external limiting membrane (ELM); the inner segment ellipsoid zone (EZ, previously called the junction between the inner and the outer segments (IS/OS junction)); the cone outer segment tips (COST) or interdigitation zone (IZ); and the retinal pigment epithelium (RPE) [5] (Figure 1). The photoreceptor outer segments (PROS) length was considered as the distance between the EZ and the RPE. Despite predominantly involving the inner retinal layers, cystoid macular edema (CME) has been treated as a separate entity.

Finally, report on vessel density (VD) changes and the FAZ area modifications in patients with MH undergoing surgery, investigated by means of OCTA, have been included in the present review. The generally accepted classification in superficial capillary plexuses (SCP), deep capillary plexuses (DCP), and choriocapillaris (CC) has been maintained.

## 3. Inner Retinal Layers

ILM peeling during vitrectomy has become a routine surgical procedure for the treatment of idiopathic MH, as the procedure significantly increases the MH closure rate and lowers the recurrence rate [18]. ILM is the basement membrane of Müller cells, the inner barrier of the neural retina, and anatomically adjacent to RNFL and GCL. Functionally, delay in the recovery of the b-waves of focal macular electroretinograms has been described; [19] anatomically, changes in the inner retinal layers thickness and alterations of their architecture correspond to the functional impair after ILM removal in MH surgery.

Based on the shape of the inner foveal layers and their tomographic contour, four macular hole closure types have been distinguished: a *U*-shape closure type, described with a contour similar to that of the healthy fovea; a *V*-shape closure type, described as a steep foveal outline; an irregular closure type, presenting as a closed hole that cannot be defined either as *U*-shape or as a *V*-shape closure type; and a flat/open closure type, described as having flat borders of the macular hole with bare RPE. Better postoperative visual acuity statistically correlates with the U-shape closure [20].

Based on the thickness of the inner foveal layers, reduction in the width of the inner retinal tomographic bands has been described by different authors [21, 22]. Kumagai et al. found that the four inner Early Treatment Diabetic Retinopathy Study (ETDRS) sectors showed a significant reduction in the average retinal thickness at 1 month, and this damage was progressive throughout the 24 months after surgery, with exception of the nasal sector [23]. From the functional point of view, a significantly positive correlation between the foveal thickness at 1 month and the visual acuity at 12 months after MH surgery has been reported. This must be interpreted that a thicker fovea in the early postoperative phase may indicate a higher degree of filling with neuronal tissue, and, therefore, a major chance of MH defect closure [24].

The changes in macular retinal thickness are not uniformly distributed at the posterior pole but rather involve specific macular quadrants and certain retinal layers. In detail, the temporal quadrants show the most severe thinning, followed by the superior and the inferior ones; conversely, in the nasal quadrant, the global thickness often increases [22, 25].

There is general consent that retinal thinning involves primarily the RNFL and GCL, with a relative sparing of the outermost retinal layers. Sabater et al. presumed that a mechanical damage due to ILM peeling involving the GCL (the layer in its whole or only the Müller cells within the GCL) is the main responsibility of these tomographic findings [26]. Faria and associates found that 6 months after ILM peeling, the RNFL, GCL, and IPL had a decreased thickness in both the nasal (−12.8 *μ*m) and the temporal regions (−29.6 *μ*m). As a possible explanation, the authors hypothesized that, after ILM peeling, inner retinal cells were particularly affected by local inflammation, microcirculatory ischemia, and stretching effects [27]. The same group found a shortening of papillofoveal distance and thickening in the outer retinal layers (ORLs) in both nasal and temporal regions during the follow-up [28].

**Figure 2:**
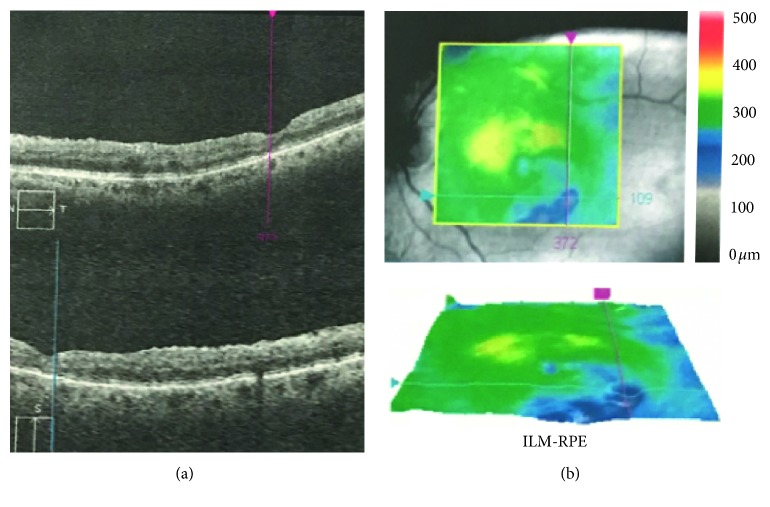
Optical coherence tomography (OCT) of dissociated optic nerve fiber layer (DONFL). B-scan OCT (a) illustrates the presence of a localized defect in the retinal nerve fiber layer and the underlying ganglion cell and inner plexiform layers in a patient who underwent macular hole repair surgery with internal limiting membrane peeling. A strong association with ILM peeling has been proved by different reports. (b) The color-coded altitude map shows a relative thinning of the retinal layers.

Qualitatively, more than 50% of the eyes that underwent successful MH repair surgery developed either superficial or deep structural alterations in the extrafoveal retina [29]. These alterations appeared on fundus photography as arcuate, slightly dark, extramacular striae along the course of optic nerve fiber and on OCT as dissociated optic nerve fiber layer (DONFL) (Figure 2) [30, 31].

Further studies have characterized DONFL with en face OCT, as multiple dark dots along the course of RNFL, called concentric macular dark spots (CMDSs), which can be eventually associated with localized defects in the underlying GCL-IPL [32, 33]. A strong association with ILM peeling has been proved: DONFLs do not develop in eyes without ILM peeling and are detectable only in the areas where the ILM peeling was performed. On the contrary, the dye used for the peeling (indocyanine green, triamcinolone acetonide, or/and trypan blue) does not seem to affect the prevalence of these lesions [34]. DONFLs appear between 1 and 3 months after ILM removal and progressively increase in number and proportionate area in the subsequent 3 to 6 months after surgery; in general, no new cases are usually observed beyond 6 months. Increase in the first months has been related to progressive deturgescence of the RNFL in the postoperative period [35].

DONFL-related defects tend to locate majorly in the temporal macular quadrant [36] and to progress with time [37, 38] but do not affect the visual acuity or the macular function [29, 39]. Earlier investigations suggested that the DONFL appearance is not due to injury to the retinal nerve fibers, but to the Müller cells, causing a cleavage in the retinal nerve fiber bundles [30].

## 4. External Retinal Layers

Physiologically, the outer retina appears on OCT as four separate bands, that correspond, starting from inside to outside, to: (1) the ELM; (2) the boundary between the ellipsoid portion of the IS; (3) the OS tips/RPE junction (contact cylinder); (4) the RPE, Bruch's membrane, and the choriocapillaris. The integrity of each of these bands, especially of bands 2 and 3, is fundamental for effective signal transmission and, in the end, for visual acuity. The prognostic relationship between preoperative morphological features of these external retinal layers and postoperative visual outcome in patients with MH has long been established. Poorer preoperative and postoperative best-corrected visual acuity (BCVA) has been correlated with larger horizontal MH basal diameter and larger preoperative diameter of the EZ defect [40].

In the postoperative period, the ELM, EZ, and COST line got restored in 100%, 69%, and 17% of eyes, respectively, after successful hole closure according to a prospective clinical series recently published [25]. This restitution is slow but progressive and takes place in the first 6 months after the surgical procedure [20, 41]; before the process gets completed, three different tomographic signs can be recognized in the foveal area.

First, a foveal detachment can persist in the first months in up to 43% of closed MHs [42], similar to that one occurring in spontaneous closure of traumatic MHs, suggesting that bridging of the inner neuroretinal tissue may be the initial step in MH repair. Secondarily, outer foveolar defects (OFDs), also called “outer retinal defects,” “foveolar lucencies,” or “foveolar cysts,” can be transiently identified in the first postoperative stage and have been associated with smaller preoperative MH [43, 44]. Once detected, the outer foveolar defects disappear, at the earliest, in 1 month, and the main time of defect disappearance is of 183 days after the surgery. Recently, the development of these lesions has been interpreted as a normal state of recovery after MH repair, associated with a more favorable surgical outcome and less-advanced preoperative MH stage [45].

Finally, a foveal hyper-reflective lesion can be noticed, which has been interpreted as a cluster of proliferative glial cells (Müller cells or astrocytes), reapproximating the normal photoreceptors to the central fovea. In different studies, the maintenance of this tissue at longer follow-up has been associated with worse visual recovery [46]. On the basis of their size, these lesions can be divided into two groups: those with larger diameter replacing the entire intraretinal foveal defect, and those with smaller diameter localized in the inner foveal layers only [47]. According to Wakabayashi and associates, the type of foveal hyper-reflective lesion is able to influence the postoperative centripetal reapproximation of the ELM on SD-OCT. If the bridging of the ELM advances faster than glial cell proliferation into the foveal defect, the hyper-reflective line corresponding to the ELM will restore. Otherwise, if the foveal defect is filled by the proliferating glial cells before the bridging of the reapproximating ELM takes place, subsequent reestablishing of the normal tomographic external retina profile at the central fovea is impeded [48]. The authors conclude that only eyes with an intact ELM have a higher chance of achieving subsequent restoration of the EZ postoperatively, and therefore, the ELM rather than the EZ acts as a critical structure predictive of the restoration potential of the foveal photoreceptor integrity in surgically closed MHs.

The relationship between the integrity of the foveal microstructure, and especially of the EZ and the ELM, and the foveal sensitivity after MH surgery has been studied by different groups (Table 2). Timely recovery of these specific layers is associated with better postoperative BCVA [14, 46, 48, 49]. In particular, the restitution of the ELM had a superior postoperative functional prognosis and acted mostly as an indirect sign of functional recovery of the foveal photoreceptors in surgically closed MHs, regardless or in association with the restoration of the EZ [48, 50, 51]. The integrity and the length of the foveal COST line were also identified as important predictors of the final BCVA: the COST line recovery begins in the peripheral region and progresses toward the center of the closed macular hole even though not always symmetrically [49, 52]. In parallel to COST line defect reduction, the BCVA progressively increases postoperatively (correlation significant at 1, 3, 6, 9, and 12 months after surgery) [53].

As far as it concerns the RPE, atrophic changes have been found both within the area of the previous MH and outside the fovea. In the former case, foveal RPE atrophy has been related to the direct contact of indocyanine green or trypan blue dye on the RPE through the retinal defect; atrophic changes outside the macula can be secondary to initial ILM incisions with the microvitreoretinal blade [54]. Engelbrecht and his group advocated that the use of minimum indocyanine green concentration and shorter time to allow for adequate staining of the ILM would reduce the potential risk for toxicity to the RPE [55]. Finally, some authors have hypothesized that the usage of the inverted internal limiting membrane (ILM) flap technique for large MH could be complicated by the expansion of submacular RPE atrophy at the long-term follow-up [56].

## 5. Cystoid Macular Edema

Postoperative CME is a major cause of visual impairment after ocular surgery. Almost any intraocular procedure can be complicated by the development of CME, including surgery for cataract, glaucoma, and cornea and vitreoretinal disorders. However, while many advances have been made in our knowledge regarding the incidence, predisposing factors, visual outcomes, and therapeutic options for CME after cataract surgery, less information is available for CME after vitreoretinal surgery [57].

Any sign of CME has been observed by structural OCT in 47% of eyes undergoing vitrectomy for nonemergent vitreoretinal indications, including epiretinal membranes, MH, vitreous hemorrhage, vitreous opacity, or tractional retinal detachment [58]. CME was more common in eyes undergoing vitrectomy for epiretinal membranes as compared to MH or nonclearing vitreous hemorrhage (incidence rate of 64% versus 29% and 29%, respectively). These significant differences can be partly explained by the tractional forces associated with epiretinal membranes, which may require more time for a full recovery, and by the different surgical and postoperative management. Also, the technique used to detect the presence of CME is important to compare the results of different studies [59].

**Figure 3:**
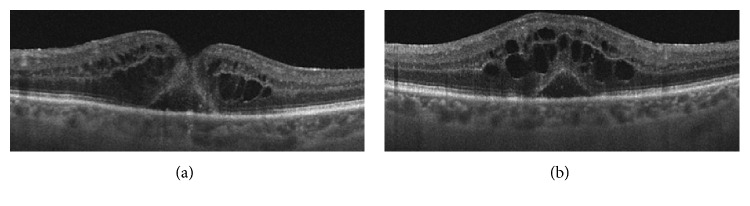
Optical coherence tomography (OCT) of macular edema following macular hole repair surgery. (a, b) Persistent hyporeflective intraretinal cyst after vitrectomy with internal limiting membrane peeling for MH.

Surgical trauma with subsequent ocular inflammation has a major role in the development of this postoperative complication, which can be suspected on fundus examination as an abnormal foveal reflex associated with a variable degree of visual disturbances. However, subtle forms of CME cannot be easily recognized without additional exams. OCT is a simple and noninvasive way to identify and monitor the presence of CME, which appears as hyporeflective cysts predominantly located in the parafoveal area (Figure 3). In more severe cases, OCT reveals the presence of a serous retinal detachment, usually of a scarce entity. The incidence rate of CME is as high as 80% of patients operated for MH far, according to the studies based on fluorescein angiography; studies based on OCT suggest an inferior prevalence of this entity.

Different factors may contribute to explain this discrepancy; it is possible that not all the vascular changes seen after vitreoretinal surgery lead to significant retinal thickening and intraretinal cysts formation. However, OCT provides more objective and reproducible measurements and can be particularly useful for assessing changes during the follow-up and for monitoring the response to treatment. Sacconi et al. observed that the main alterations in CME after cataract surgery were located at the level of the deep capillary plexus and were partially reversible after therapy [60].

Different therapeutic options have been used in postsurgical CME. Topical treatment with nonsteroidal anti-inflammatory drugs (NSAIDs) and corticosteroids aims at decreasing the production of inflammatory molecules involved in the breakdown of the blood-retinal barrier. Topical NSAIDs have been shown to be able to reach the vitreous chamber in significant concentrations to produce structural and clinical changes [57]. Systemic steroids, on the opposite, were not able to give significant functional and anatomical improvements in patients with postsurgical CME after vitreoretinal procedures. For refractory cases of CME after vitrectomy, sustained-release dexamethasone intravitreal implant proved to be useful in different studies. This treatment was effective by improving visual acuity by one or more Snellen lines in most of the treated eyes, with effect duration up to 9 months. Functional results after a single injection of dexamethasone implant correlated with a significant decrease of mean central retinal thickness measured by structural OCT [61]. Another treatment option described in cases of recalcitrant CME includes the use of intravitreal injection of triamcinolone acetonide. This treatment was effective in reducing CME, but the visual improvement was transient. In persistent cases of CME, intravitreal injections of anti-VEGF have also been tried and showed no significant benefits on central macular thickness and visual improvement [62]. It is possible that intravitreal injections of anti-VEGF in vitrectomized eyes have a limited duration because of a more rapid clearance.

In conclusion, the development of CME can complicate MH repair surgery, and the noninvasive diagnosis and follow-up with OCT is a simple and fast way to identify and monitor this condition and its response to medical treatment.

## 6. Macular Perfusion

Up to the present, few pieces of evidence have been published analyzing the VD changes and the FAZ modifications in patients with MH undergoing surgery. At baseline, qualitative analysis of en face OCTA slabs shows a central, round flow defect in the SCP and DCP, surrounded by perifoveal hyporeflective pseudocysts more consistent at the DCP. These cysts appear either as large, regular, and well-defined radial cystic areas with a petaloid or “grapefruit” configuration or as small, dispersed lesions with a “sponge-like” appearance [63]. Quantitatively, the investigations of the structural preoperative SCP and the DCP have proven a diffuse compromising, more pronounced in the DCP in long-standing, large MHs and a larger FAZ compared to controls [64]. The apparent expansion or enlargement of the FAZ can be interpreted as the loss of retinal tissue at the center of the fovea happening in MHs. However, it might be related to centrifugal tractional forces acting on the foveal center; dilatation of the FAZ would be in this case the result of mechanical factors.

Postoperatively, OCTA has globally demonstrated that eyes after MH closure feature a reduction in macular and paramacular vasculature, compared with control or fellow eyes. At the same time, different authors have described, independently, a reduction in the FAZ area at both the SCP and the DCP after the surgical repair [65–67], symmetrical to the size of the fellow eye [68]. The FAZ area tends to be inversely correlated to the postoperative foveal thickness [66] and the postoperative BCVA, suggesting that eyes with smaller FAZ areas disclose a better postoperative visual outcome up to 6 months after the surgery. The reduction in FAZ size has been interpreted either as the result of the release of tractional forces after MH closure or a consequence of the centripetal dragging of retinal tissues after ILM peeling [69]. On the other hand, the link between larger FAZ and worse functional outcome is consistent with findings in retinal diseases other than MH, including age-related macular degeneration, diabetic retinopathy, or central retinal vein occlusion [70, 71]. Therefore, the FAZ area is advocated as a surrogate indicator of the neurovascular integrity of the fovea.

Similar to FAZ, also the retinal VD of SCP and DCP was associated with postoperative retinal thickness, and in particular, with mean GCL-IPL thickness [72]. An issue that has been raised with repeated FAZ and VD measurements before and after MH closure deals with macular displacement secondary to ILM peeling. Comparative analysis of preoperative and postoperative fundus photographs has shown that the fovea is generally displaced toward the optic disc after surgery, with the temporal vessels shifted more than the nasal vessels. This macular dragging nasally and inferiorly has been recently demonstrated also by means of OCTA; using the fovea as a fixed point, a centripetal displacement of the vessel bifurcations has also been confirmed [17].

**Figure 4:**
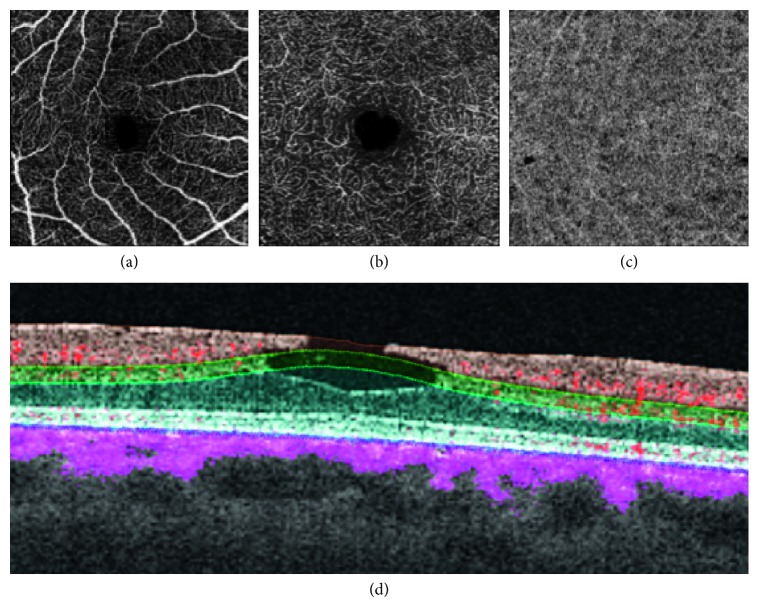
Optical coherence tomography (OCT) angiography in a case of degenerative lamellar macular hole. Superficial vascular plexus (top left panel) and deep vascular plexus (top middle panel) illustrate alterations of the foveal avascular zone, corresponding to the lamellar loss of inner retinal tissue. Segmentation at the level of the choriocapillaris (top right panel) demonstrates normal perfusion. The exact segmentation of the superficial vascular plexus, deep vascular plexus, and choriocapillaris (bottom panel) is illustrated on B-scan OCT with blood flow superimposed (red flow for retinal circulation and pink flow for choroidal circulation).

In the parafoveal region, eyes after MH surgery had a tendency to have a lower vascular density at the DCP, closely linked to the global retinal thickness in that area. This is true for generative LMH also (Figure 4) [63, 66, 73]. The explanation for this finding seems to be linked to the presence of intraretinal pseudocysts preoperatively seen at the edge of the MH. These pseudocystic areas localize between the inner nuclear layer (INL) and the outer plexiform layer (OPL), where the capillary bed of the DCP is embedded; despite a successful MH closure and anatomical reapposition of the walls of the pseudocysts, vascular atrophy corresponding to the pseudocystic cavity remains and appears as a darker hole on OCTA.

As far as it concerns the CC, Teng et al. found that the flow area and the parafoveal VD of CC in the macular area were significantly lower in eyes with MH than unaffected fellow eyes and healthy controls; this diminished CC circulation was partially restored after surgery [74]. Findings confirming CC perfusion defects were then confirmed by Ahn et al., who also observed that VD in the CC in LMH was not significantly different from that seen in fellow eyes and normal controls [75].

In conclusion, the quantitative evaluation of vascular capillary networks and of the FAZ may serve as a useful anatomic biomarker for assessment of macular perfusion parameters before and after MH repair.

## 7. Conclusions

Different devices have been recently added to the diagnostic tools available for the postoperative assessment of vitreoretinal surgery and for the prompt recognition of surgical complications. Among all these newly introduced devices, OCT and OCTA have added great value and significant benefits for the monitoring of the clinical course after vitreoretinal surgery. Different clinical findings can be observed on OCT of patients who underwent MH repair surgery, including inner retinal changes, outer retinal changes, CME, and macula perfusion abnormalities. Clinicians should be aware of these tomographic findings in order to properly manage each of the aforementioned condition. In addition, significant advantages for the postoperative application of OCT and OCTA include its noninvasiveness, rapid and simple execution, repeatability, and precise measurements. Taking together all these points, the OCT integrated into the clinical monitoring of patients after MH surgery is an invaluable diagnostic tool, critical for proper therapeutic management.

## Figures and Tables

**Table 1:** The International Vitreomacular Traction Study (IVTS) Group classification of macular hole (MH).

Size	Small (250 mm)
	Medium (>250–400 mm)
	Large (>400 mm)
Status of vitreous	With VMT
	Without VMT
Cause	Primary or idiopathic
	Secondary (caused by other pathologies, without any preexisting or concurrent VMT)

VMT: vitreomacular traction. Source: Duker et al. [11].

**Table 2:** Prognostic value of postoperative optical coherence tomography (OCT) external retinal layers after macular hole (MH) repair surgery.

OCT layer	Authors	Year	Type of study	Eyes/patients	ILM peeling (yes/no)	Dye	MH diameter (*μ*m, mean ± SD and/or range)	Mean follow-up (months)	Comment
OFD	Kang et al. [43]	2010	Retrospective	96/93	NA	NA	333.5 ± 126.3 with OFD 504.2 ± 155.6 without OFD	14.4	OFD associated with better preoperative and postoperative BCVA
	Powers et al. [45]	2018	Retrospective	104	Yes	NA	NA	NA	ORD may represent a normal state of recovery after MH repair with ILM peeling

ELM	Wakabayashi et al. [48]	2010	Retrospective	40/40	Yes	Indocyanine green dye or triamcinolone acetonide	623 ± 303 (144–1235)	12	Reconstruction of ELM at 3 months associated with better BCVA at 3 and 12 months
	Bottoni et al. [44]	2011	Prospective	19/19	Yes	Indocyanine green or brilliant blue G	NA	12	Combined recovery of ELM, EZ, and ONL determined VA improvement, but ELM was first structure to recover after MH closure
	Ooka et al. [50]	2011	Prospective	43/43	Yes	Indocyanine green dye or triamcinolone acetonide	NA	6	Length of both EZ and ELM defects significantly correlated with postoperative BCVA and foveal sensitivity

EZ	Sano et al. [42]	2009	Retrospective	28/27	Yes	Indocyanine green	NA	7.7	EZ was the only relevant factor affecting postoperative BCVA at six months
	Michalewska et al. [20]	2010	Retrospective	71/66	Yes	Trypan blue	666–1386	12	BCVA correlated with EZ. 93% had EZ defects at 1 week; only 29.5% had EZ defects at 12 months
	Oh et al. [46]	2010	Retrospective	23/23	Yes	None or indocyanine green dye or triamcinolone acetonide	104–998	3	Larger diameter of EZ defect and apparent glial sealing correlated with worse postoperative BCVA
	Chang et al. [51]	2015	Retrospective	60/56	Yes	Indocyanine green	NA	12	Postoperative BCVA correlated with restored ELM and EZ line and resolved glial cells

COST or PROS length	Itoh et al. [53]	2012	Retrospective	51/51	Yes	Indocyanine green dye or triamcinolone acetonide	336 ± 152 (136–946)	12	Preoperative length of COST line defect correlated with postoperative BCVA at 12 months
	Hashimoto et al. [49]	2015	Retrospective	24/23	Yes	Triamcinolone acetonide	NA	28.7	Postoperative BCVA correlated exclusively with foveal PROS elongation

SD: standard deviation; NA: not assessed; OFD: outer foveolar defects; ELM: external limiting membrane; EZ: ellipsoid zone; COST: cone outer segment tips; PROS: photoreceptor outer segment.
